# Comprehensive Management of Cocaine-Induced Midline Destructive Lesions: A Young-IfOS Consensus

**DOI:** 10.3390/jpm15060231

**Published:** 2025-06-03

**Authors:** Alberto Maria Saibene, Letizia Nitro, Florent Carsuzaa, Mihaela Alexandru, Vincent Bedarida, Matteo Di Bari, Léa Fath, Ainhoa Garcia-Lliberos, Margaux Legré, David Lobo-Duro, Antonino Maniaci, Thomas Radulesco, Leigh Sowerby, Neil Tan, Manuel Tucciarone, Clair Vandersteen, Valentin Favier, Maxime Fieux

**Affiliations:** 1 Young Otolaryngologists-International Federation of Otorhinolaryngological Societies (Yo-IFOS), F-75000 Paris, France; alberto.saibene@gmail.com (A.M.S.); letizia.nitro@gmail.com (L.N.); florent.carsuzaa@gmail.com (F.C.); mihaela.dana.alexandru@gmail.com (M.A.); vincent.bedarida@gmail.com (V.B.); mattediba@gmail.com (M.D.B.); lea.fath@chru-strasbourg.fr (L.F.); ainhoa.glliberos@gmail.com (A.G.-L.); margauxlegre@gmail.com (M.L.); david.lobo@scsalud.es (D.L.-D.); tnmaniaci29@gmail.com (A.M.); thomas.radulesco@gmail.com (T.R.); leigh.sowerby@gmail.com (L.S.); neil.tan@nhs.net (N.T.); tucciaronemanuel@hotmail.it (M.T.); vandersteen.c@chu-nice.fr (C.V.);; 2 Otolaryngology Unit, ASST Santi Paolo E Carlo, Department of Health Sciences, Università Degli Studi Di Milano, F-20133 Milan, Italy; 3 Service ORL, Chirurgie Cervico-Maxillo-Faciale et Audiophonologie, Centre Hospitalier Universitaire de Poitiers, F-86000 Poitiers, France; 4 Laboratoire Inflammation Tissus Epithéliaux et Cytokines (LITEC), Université de Poitiers, 86000 Poitiers, France; 5 Service d’Orl et Chirurgie Cervico-Faciale, Assistance Publique-Hôpitaux de Paris (AP-HP), Université Paris-Saclay, Hôpital Bicêtre, F-94270 le Kremlin-Bicêtre, France; 6 Sorbonne Université, Institut National de la Santé et de la Recherche Médicale, Hôpital Armand-Trousseau, F-75012 Paris, France; 7 Otolaryngology-Head and Neck Surgery Department, Hôpital Lariboisière, Assistance Publique-Hôpitaux de Paris, F-75010 Paris, France; 8 Otolaryngology Department, Hôpitaux Universitaires Pitié Salpêtrière-Charles Foix, Assistance Publique-Hôpitaux de Paris, F-75013 Paris, France; 9 Service d’ORL, de Chirurgie Cervico Faciale, Hôpital de Hautepierre, Hôpitaux Universitaires de Strasbourg, F-67200 Strasbourg, France; 10 Unité Inserm 1121, Biomatériaux et Bioingénierie, F-67000 Strasbourg, France; 11 ENT Department, Valencia General University, F-46014 Valencia, Spain; 12 Service ORL et Chirurgie Cervico-Faciale, Institut Arthur Vernes, F-75006 Paris, France; 13 Otolaryngology Department, Hospital Universitario Marques de Valdecilla, F-39008 Santander, Spain; 14 Valdecilla Biomedical Research Institute (IDIVAL), F-39008 Santander, Spain; 15 Department of Otolaryngology, Kore University, F-94100 Enna, Italy; 16 ENT-Head and Neck Surgery Department, APHM, La Conception University Hospital, Aix Marseille University, F-13005 Marseille, France; 17 CNRS, IUSTI, Aix Marseille University, F-13007 Marseille, France; 18 Department of Otolaryngology-Head and Neck Surgery, Western University, London, ON N6A 3K7, Canada; 19 Department of Otolaryngology, Royal Cornwall Hospital, Truro TR1 3LJ, UK; 20 Department of Otolaryngology and Head and Neck Surgery, University Hospital of Jerez, F-11407 Cádiz, Spain; 21 Institut Universitaire de La Face et du Cou, Centre Hospitalier Universitaire de Nice, F-06000 Nice, France; 22 Unité de Recherche Clinique de Nice Côte d’Azur (UR2CA), Université Côte d’Azur, F-06000 Nice, France; 23 Département d’ORL, Chirurgie Cervico Faciale et Maxillo-Faciale, Hôpital Gui de Chauliac, CHU de Montpellier, F-34295 Montpellier, France; 24 Research-Team ICAR, Laboratory of Computer Science, Robotics and Microelectronics of Montpellier (LIRMM), University Montpellier, French National Centre for Scientific Research (CNRS), F-34295 Montpellier, France; 25 Hospices Civils de Lyon, Centre Hospitalier Lyon Sud, Service d’ORL, D’otoneurochirurgie et de Chirurgie Cervico-Faciale, F-69495 Pierre Bénite, France

**Keywords:** endoscopy, computed tomography, recreational cocaine use, differential diagnosis, granulomatous disease, cocaine-induced midline destructive lesion

## Abstract

**Background:** Recreational nasal cocaine use (RNCU) presents a significant challenge for rhinologists due to cocaine-induced midline destructive lesions (CIMDLs). This clinical consensus statement (CCS) offers guidelines for diagnosing, assessing, and managing both proven and suspected cases of CIMDL (including those without a prior RNCU history). It aims to support clinicians in addressing these complex cases effectively. **Methods:** An international, multidisciplinary panel of 18 specialists employed a three-round modified Delphi-method survey to evaluate statements covering CIMDL management issues such as definition, clinical evaluation and diagnosis, initial management approaches, and surgical management of complications and reconstructions. This study primarily targets otorhinolaryngologists. **Results:** Out of 44 evaluated statements, 20 achieved strong consensus, 20 reached consensus, 3 approached near-consensus, and 1 failed to achieve consensus. Consensus-covered areas included the definition of CIMDL, clinical evaluations, first-line management, and management of complications. However, reconstructive techniques remained a contentious topic. **Conclusions:** In the absence of extensive data, this CCS establishes a management framework for CIMDL, significantly bridging a knowledge gap. It highlights the need for standardized assessments, multidisciplinary cooperation, and customized follow-up care for patients with CIMDL. Considering the widespread use of cocaine, physicians should consistently consider the possibility of RNCU when encountering chronic inflammatory lesions in the sinonasal tract.

## 1. Introduction

Estimating the prevalence of cocaine snorting, or recreational nasal cocaine use (RNCU), is complex due to its illegal status and potential survey underreporting. The United Nations Office on Drugs and Crime’s World Drug Report 2021 estimated that around 19 million people globally, or 0.4% of the population aged 15–64, used cocaine in any form during 2019 [1]. In Europe, the European Monitoring Centre for Drugs and Drug Addiction reported that 2.3% of individuals aged 15–34 years used cocaine in 2022 [2]. The lifetime prevalence of RNCU is around 3.9% in Brazil, with European rates varying from 0.3% to 11% [2,3]. This makes RNCU notably significant due to the array of potential health symptoms it can induce.

Chronic RNCU (defined as use for at least 6 months, administered at least 4 times a month) can lead to severe health issues, including hyperthermia, cardiovascular problems, and neurological disorders [4], significantly affecting patients’ quality of life [5]. From a rhinological perspective, RNCU can cause vasoconstriction and damage to the nasal mucosa, progressively harming the nasal perichondrium and periosteum. This often results in a damaging condition known as cocaine-induced midline destructive lesions (CIMDLs) [6]. Adulterants in cocaine, such as levamisole, may exacerbate these destructive effects through autoimmune responses [6,7]. CIMDLs can vary from minor septal perforations to severe skull base damage, with the Nitro et al. classification system developed to categorize these injuries [5]. Early signs like septal perforations (Nitro et al. classification grade 1) often present diagnostic challenges as they are not specific to RNCU [8].

Given the scarcity of research specifically addressing the diagnosis, assessment, and management of CIMDL, predominantly retrospective, an expert-led clinical consensus statement (CCS) has been developed. This CCS utilizes a modified Delphi process to provide management guidelines based on the best available evidence for proven and suspected CIMDL, addressing common and complex clinical scenarios.

## 2. Materials and Methods

This CCS was developed according to the modified Delphi protocol by Rosenfeld et al. [9]. The French Ethic Committee of otorhinolaryngology has issued a favorable decision (n° 2024-03-035-VF) to conduct the study.

### 2.1. Panelists and Scope of Consensus Statement

The Delphi committee was made up of 18 voluntary members of the Young Otolaryngologist section of the International Federation of Otorhinolaryngological Societies (Yo-IFOS) rhinology research group from five countries across Europe and North America. The YO-IFOS research group is an invitation-only group, whose members are selected by the elected scientific committee of the YO-IFOS among worldwide board-certified otolaryngologists younger than 45 years old based on the extent and impact of their scientific production. The group is further subdivided according to members’ subspecialties. As is the case for this CCS, members are free to propose new projects and participate in proposals according to their areas of expertise (though one completed proposal and two participations every 24 months are required to retain membership). The core development team included a chairperson (VF), a vice-chairperson (LN), and a methodologist (AMS). VF and LN were selected as leading and proposing the research project, and AMS due to their extensive experience both in CIMDLs and in Delphi consensuses. An additional team comprising two rhinologists randomly chosen among panelists (FC and MF) enhanced the initial draft of the CCS. These rhinologists joined on a voluntary basis from the Yo-IFOS or were recommended by the group as experts in CIMDLs. The authors reported no conflicts of interest. The CCS was designed to provide specific recommendations for managing CIMDLs.

### 2.2. Systematic Literature Review

An equation research systematic review adhering to PRISMA guidelines was carried out across the MEDLINE, EMBASE, Scopus, and Web of Science databases to explore management strategies for CIMDLs. Comprehensive search terms including both the acronym and full definition of CIMDLs, along with ‘cocaine’ and related terms pertaining to the paranasal sinuses and nose, were employed on 19 July 2023. This search aimed to identify studies published in English, Italian, German, French, or Spanish that presented data derived from human participants.

The basic search string was (cocaine and (midline OR nose OR turbinate OR concha OR nasal OR palate OR “skull base” OR sinus OR septum)) OR CIMDL OR “cocaine-induced midline destructive lesion”.

Owing to the scarcity of high-quality research available, the scope of the systematic review was broadened beyond the initially advised parameters for CCSs, which typically encompass guidelines and systematic reviews [9]. It was expanded to incorporate randomized clinical trials relevant to the subject, adopting a methodological approach that aligns with other recent CCSs in the field of head and neck research [10,11].

**Figure 1:**
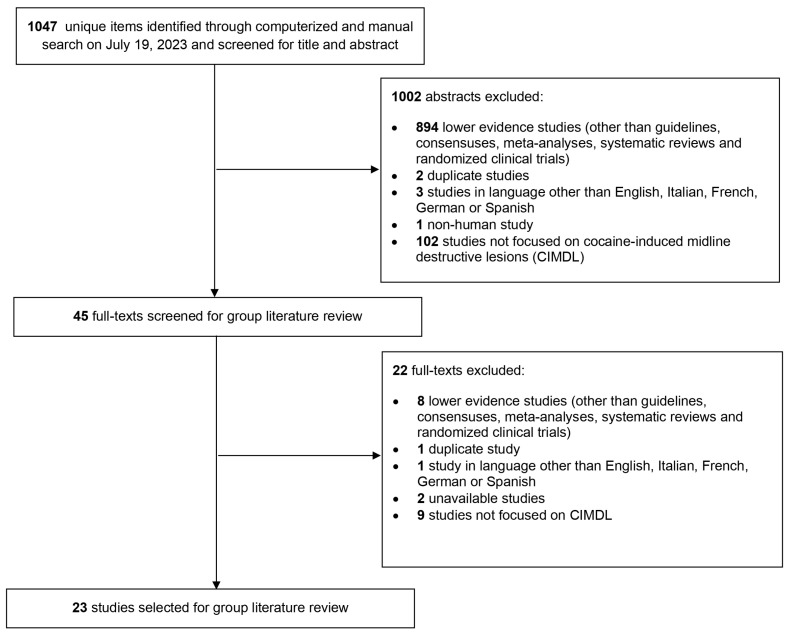
PRISMA-style flow chart of the article selection process for the systematic review of the literature about cocaine-induced midline destructive lesion (CIMDL) management.

Based on the search equation and inclusion criteria set up initially to perform a systematic review, 23 articles were included [5,6,12,13,14,15,16,17,18,19,20,21,22,23,24,25,26,27,28,29,30,31,32], representing the highest-quality evidence available on the subject. However, as most of the studies were only descriptive low-level-of-evidence (level 4 or 5) studies without any control group, a systematic review could not be performed, justifying the choice of a CCS methodology. This compilation was shared with all authors for review over a period of one month. Figure 1 illustrates the selection process of these articles using a PRISMA flow chart. The authors involved were invited to suggest any additional literature they deemed crucial to the CCS’s breadth. Consequently, one more article was added to this collection [33].

### 2.3. Clinical Statement Development and Modified Delphi Survey

Guided by the literature review and the objectives of the CCS, the chair and assistant chair crafted the initial clinical statements for the survey. These statements were then elaborated, refined, and extended through discussions with the methodologist. Following this, the secondary development group evaluated the preliminary draft prior to initiating the Delphi rounds.

The statements were shaped by insights from the literature review and the development group’s understanding of key clinical scenarios, resulting in a comprehensive 44-statement survey. This survey was disseminated to the authors via Google Forms (Google LLC, Mountain View, CA, USA). Participants were asked to fill out the survey anonymously, using a one-time link and a personal identification number (PIN) to prevent duplicate responses and maintain anonymity. The coordination of PINs was managed by a single coordinator, who was not directly involved in the CCS, ensuring the anonymity of panelist responses. The authors rated each statement on a 9-point Likert scale ranging from strongly disagree (1) to strongly agree (9). According to Rosenfeld [9], the outcomes for each statement were categorized as follows: strong consensus was achieved if the mean score was ≥8.00 without any outliers (an outlier being a rating that deviated by 2 or more points from the mean in either direction); consensus was noted if the mean score was ≥7.00 with no more than one outlier; near-consensus was indicated by a mean score of ≥6.50 with no more than two outliers; all other responses were categorized as having no consensus. All panelists were asked to provide comments explaining their refusal or proposing new statements to be implemented in the following rounds.

## 3. Results

All panelists participated in three Delphi rounds. After the initial round, consensus levels were as follows: 4 out of 44 statements achieved strong consensus, 9 reached consensus, 16 reached near-consensus, and 15 did not reach consensus. The 31 statements that did not achieve at least consensus were revised for greater inclusivity and clarity, based on anonymous feedback from the authors. The revised second round included these 31 statements, resulting in 5 achieving strong consensus, 6 reaching consensus, 10 achieving near-consensus, and 10 still lacking consensus. Following further revisions, a third and final round was conducted with 20 statements, of which 11 statements achieved strong consensus, 5 reached consensus, 3 reached near-consensus, and 1 did not achieve consensus. Overall, from the total of 44 statements, 20 reached strong consensus, 20 reached consensus, 3 reached near-consensus, and 1 failed to reach consensus after all rounds. The progression of the statements from the first round to their finalized versions is detailed in Appendix A. The results of the Delphi process for all statements, including their mean scores, median scores, score ranges, and the number of outliers, are documented in Table 1, Table 2, Table 3, Table 4 and Table 5. Definitions of CIMDLs are shown in Table 1, clinical evaluation and diagnosis in Table 2, first-line management in Table 3, and surgical management of complications in Table 4, while statements with no consensus are in Table 5.

The top-scoring items for strong consensus were “Patients with CIMDL should be correctly informed that no “safe threshold” of cocaine usage prevents further CIMDL evolution” (mean score 8.89, median 9, no outliers) and “CIMDL can refer both to sinonasal and palatal lesions due to cocaine-snorting” (mean score 8.83, median 9, no outliers). A total of 29 out of 44 items recorded a median score of 9.

The lowest-scoring items, with mean scores of 7.61 and 7.44 and a median score of 7.5 for both, stated “CIMDL are due to RNCU only, while they are not related to nasal cocaine medical administration” and “CIMDL are defined as any structural lesion of the sinonasal complex ascertained in the context of a toxicological screening- or patient history-confirmed cocaine-snorting habit”. The first of these two items was eventually removed from the CCS, while the second reached consensus.

## 4. Discussion

**Figure 2:**
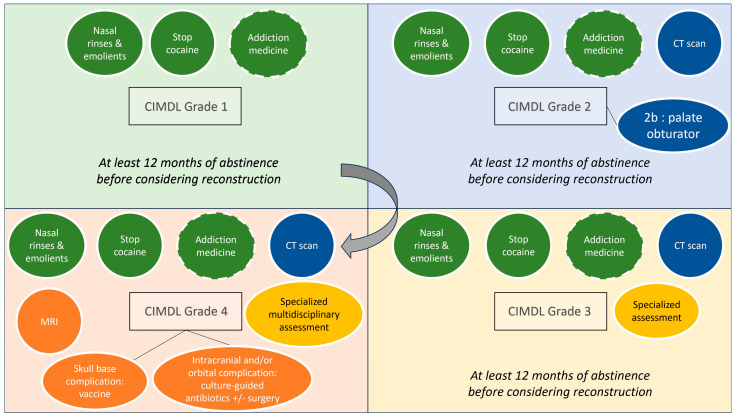
PRIMSA flow chart of the literature. Overview of the cocaine-induced midline destructive lesion (CIMDL) management proposed according to Nitro et al.’s classification grades. Dotted circles represent options to discuss with patients on a case-by-case basis (CT, computed tomography; MRI, magnetic resonance imaging).

The multidisciplinary group of experts involved in the creation of this Delphi-method consensus statement has delineated a specific all-around management guideline for CIMDLs, thereby filling an important gap in the literature. Nitro et al. [5] proposed a classification system for CIMDLs according to the severity of the disease, which has been accepted by the panel. However, this initial article did not provide guidance on CIMDL management. Despite the scarcity of solid evidence, this CCS aims to improve outcomes and define a basic standard of care for CIMDL patients, focusing on complex diagnoses and recurrences. Therefore, this CCS can guide general otolaryngologists in managing challenging cases and allied specialties in referring patients for rhinology evaluation. A common need for standardization of CIDML care is evident from the frequent outliers in our peer-reviewed consensus statements, even in the disease definition sections. An overall management framework graphic representation based on the Nitro et al. classification of CIMDLs is reported in Figure 2.

### 4.1. Disease Definition

According to the panel, CIMDL is a clinical diagnosis based on the presence of at least one sinonasal lesion and a patient-confirmed history of RNCU. This resolution represents a significant step towards simplifying the diagnosis. It follows more modern views on the subject [6,31], in contrast to older positions that required at least two lesions for CIMDL diagnosis [34]. According to our CCS, there is no need to perform systematic auto-antibody testing when the clinical history is clear, given the inconclusive significance of a positive test in RNCU patients [6,35,36,37]. RNCU-induced mucosal membrane alteration leads to CIMDLs, possibly affecting the palate, the sinonasal complex, and its neighboring structures such as the orbits or the skull base [30,38]. This potential involvement justifies performing a complete facial, nasal, and oral examination and the use of anterior rhinoscopy and nasal endoscopy. Interestingly, the CCS failed to reach a consensus threshold regarding the use of medical nasal cocaine administration as a cause of CIMDLs. Medically administered cocaine has a known risk of inducing nasal lesions [18], but at present, CIMDLs should remain confined to RNCU patients, given the peculiar disease course. It is worth remembering that the recommended therapeutic topical dose of cocaine for local anesthesia is 1.5 mg/kg [39], and there is a 35% systemic absorption of topical nasal cocaine use [40], with some reports of cardiac complications following medical nasal administration [41]. By contrast, such a dose is far from RNCU exposure, with daily “runs” of 1000 mg and over [26], while recent studies have proven efficacy and safety profiles when medical cocaine is properly administered [42]. It is worth noting that medical cocaine use is declining in most regions [43], further reducing the potential for iatrogenic cocaine-induced lesions. A challenge remains for the definition of CIMDLs when patients have withdrawn from any RCNU in the past few months. When facing an isolated septal perforation, both the duration of the abstinence from drug use and the clinical characteristics of the midline lesion should be considered by physicians as time only is not reliable enough. As suggested in statements 2.3, 2.4 and 2.5, if there was RCNU within the previous years but without any recent consumption, the observation of an isolated septal perforation with good healing (neither crusting nor inflammation) could suggest a CIMDL. However, if there was active inflammation or crusting regarding the perforation, then further explorations should be run and a differential diagnosis should be considered.

### 4.2. Clinical Evaluation and Diagnosis

Given its scientific background and the lack of other significant options, our CCS suggests using the Nitro et al. classification for grading CIMDLs. This classification was developed according to a literature review to standardize the report of sinonasal and palatal anatomical structure involvement [5]. It describes four stages of increasing CIMDL anatomical structure involvement: nasal septum (grade 1), inferior part of the lateral nasal wall and hard palate (grade 2a and 2b), ethmoid and sphenoid region (grade 3), and orbit or skull base involvement (grade 4).

Differential diagnosis is one of the most difficult aspects of CIMDL management explored in this CCS. In a considerable number of cases, midline destructive lesions are not linked to a declared history of cocaine snorting and/or caused by RNCU [44]. According to the CCS, a patient with grade 2 or higher CIMDL with no history of drug snorting should undergo a complete workup to search for autoimmune, infection, or tumoral lesions using serology (ESR, ANA, C-ANCA—with immunofluorescence for NPO and PR3 if positive—IgG4, and rheumatoid factor), rheumatology/internal medicine and infectious disease consultation, and nasal biopsy. Also, differential diagnosis should always include RNCU even if the patient initially denies it. In these cases, drug tests may be performed on-site using urine tests or hair tests based on the suspected delay between the last consumption and the visit. This diagnostic strategy could also be applied in case of enlarging septal perforation during the follow-up because idiopathic midline lesions are rare [27]. To improve sensibility, panelists proposed to perform a biopsy, either under local or general anesthesia according to surgeons’ and patients’ preferences. It is important to remember that any crusted lesion of the nasal mucosa may be cancer, but some lesions such as primary sinonasal lymphoma can be challenging to diagnose even at the biopsy level and often require collecting multiple samples [45].

For patients presenting with non-otherwise-explained rhinitis [46] or an isolated septal perforation (grade 1 equivalent in the Nitro et al. classification), RNCU should be investigated as well as the use of intranasal vasoconstrictors, picking habits, and traumatic, infectious or autoimmune history. Of course, when there is any suspicion of tumoral involvement in the mucosa, a biopsy should be performed. The choice of cocaine tests (urinary metabolite analysis and/or hair analysis) in patients with potential CIMDLs has been debated and only achieved a near-consensus in the third CCS round [47], with panelists questioning the timing, modalities, and appropriateness of tests. Cocaine testing was proposed to confirm CIMDLs in patients who denied RNCU [48]. While this approach could be used on a case-by-case basis to offer patients a targeted support approach to recover from addiction, it is important to remember that rare false positives exist and might lead to wrong medical decisions [49]. Interestingly, levamisole can also be detected in urine tests, and it could be relevant to include it in the drugs usually screened to assess the risk of induced vasculitis [50]. In the case of non-cocaine-induced midline lesions, other recreational nasal substance use should be assessed (e.g., heroin [51] or methamphetamine [52]) and—if present—managed in a CIMDL-like framework.

At the other end of the severity spectrum, our CCS suggests that grade 3–4 CIMDL patients should be assessed for unknown orbital and skull base complications at presentation, as these patients require a specialized consultation with multidisciplinary clinical evaluation [53].

The use of a plain computed tomography (CT) of the sinuses was recommended in our CCS for CIMDL grade 2 patients or higher. In these cases, the CT scan can help to distinguish between grades 2–3 and 3–4 and point clinicians toward the correct specific management and follow-up plan. A CT scan with contrast should be reserved for suspected cases of osteitis/osteonecrosis, as the non-necrotic mucosa in people who use cocaine is extremely inflammatory and carries a risk of misinterpretation. Magnetic resonance imaging (MRI) must be proposed in case of orbit or skull base involvement to analyze the extension and infectious complications.

### 4.3. First-Line Management of CIMDL Patients

As CIMDLs are considered a complication of repeated RNCU, if allowed by local policies and medical facility availability, all patients should be encouraged to meet a specialist in addiction medicine for psychological [54] and pharmacological help [55]. In patients with painful symptoms due to CIMDLs, strong opioids should be avoided and intractable pain should be managed by an addiction/pain medicine service [56]. Furthermore, the CCS highlighted that there is no safe RNCU threshold and only complete RNCU cessation can block the disease progression [31]. Last, as is usually the case with complex conditions that transcend the boundaries of otolaryngological disease, our CCS suggests that follow-up should be personalized to encourage cocaine-snorting cessation.

### 4.4. Surgical Management of Complications and Reconstructions

In extreme cases, CIMDL extension can lead to orbital [57,58], skull base [38,59], or craniovertebral junction complications [60]. These potentially life-threatening infections require multidisciplinary management. When possible, the CCS favored a conservative treatment plan with nasal toilette, antibiotics for superinfection, and applying vaccine recommendations to prevent pneumococcal, Haemophilus, and meningococcal meningitis. The intracranial complications, such as infection or cerebrospinal fluid leaks, require collaboration with a neurosurgical team for a tailored surgical strategy. Skull base reconstruction after necrotic tissue debridement should be performed in ascertained cerebrospinal fluid leaks, using vascularized flaps when possible [61].

Apart from these life-threatening situations, reconstruction of a CIMDL defect—septum or palate—should only be proposed in patients with evidence of cessation of RNCU. Most groups performing reconstruction in CIMDL patients rely on repeated negative urine metabolite tests before considering surgery [62,63,64]. Our CCS recommended at least 12 months of abstinence, supported by toxicological analysis, and strong patient motivation to consider surgical repair. This threshold was suggested according to the literature where the sustained remission phase begins after 12 months of abstinence from drug use (Diagnostic and Statistical Manual of Mental Disorders fifth version criteria [65]). Indeed, evidence showed that the longer the duration of abstinence, the lower the risk of relapse. Unfortunately—but this is marginal concerning the scope of this CCS and suffers from the anecdotal nature of the pertaining literature—we failed to reach a consensus on the basic techniques that should be considered when treating CIMDL patients for reconstructive purposes.

The use of hyperbaric oxygen therapy to accelerate the pre- and postoperative reconstruction process may be an option to discuss but requires further evidence [66].

### 4.5. Limitations of the CCS

There were some limitations to the study due to its subjective nature. Authors tried to assess all relevant questions pertaining to CIMDLs, while focusing on sinonasal cavity lesions because they are responsible for almost all rhinology functional symptoms. Cocaine snorting may also affect the dorsal septum, nasal pyramid or lips with aesthetic complaints that were not assessed in the present study only involving rhinologists. A multidisciplinary CCS including input from rhinologists, maxillofacial surgeons, and plastic/reconstructive surgeons may be required to respond to the challenging management of nose deformities following cocaine snorting. However, while this CCS aims to provide guidance on common cases, it does not intend to replace the medical decision-making process, or to challenge the 12-step approach to control relapse in outpatient treatment for cocaine abuse, especially when physicians are facing heavily addicted users [67,68,69]. ENT specialists are neither experts nor referents in this field, which falls within the remit of addictology, and patients should be referred as recommended in paragraph 3.1: “CIMDL patients could be encouraged to meet a specialist in addiction medicine”. At this stage, even though this CCS is a robust first step to guide physicians, many questions remain unanswered because of the difficult relationship of trust between physicians and drug users. Indeed, most drug addicts do not declare their consumption, and relapses are frequent. Unrepentant drug addicts, but also those who have difficulty weaning themselves off, should all use nasal rinses like any non-drug user suffering from rhinology symptoms [70]. It is also important to note that even if this study only focuses on cocaine abuse, assessing for multiple drug abuse is necessary as the latter can increase the complexity of CIMDL management. In these cases, a multidisciplinary approach with a thorough addictology assessment and close follow-up should be proposed, adapting the proposed statements on a case-by-case basis.

## 5. Conclusions

CIMDLs present significant obstacles in terms of diagnosis, addiction management, and therapeutic approach. This clinical consensus statement (CCS) recommends standardizing initial assessments and implementing customized follow-up plans, which should include multidisciplinary collaboration for more complex cases. Considering the prevalent use of cocaine in the general population, physicians should routinely consider cocaine snorting as a possible cause when encountering chronic inflammatory lesions in the sinonasal tract.

## Figures and Tables

**Table 1:** Statements and results from the Delphi process for items reaching consensus or strong consensus: disease definition.

Item No.	Statement	Mean	Median	Min	Max	Outliers	Result	Delphi Round
1.1	CIMDLs are defined as any structural lesion of the sinonasal complex ascertained in the context of a toxicological screening- or patient history-confirmed cocaine-snorting habit	7.44	7.5	1	9	1	consensus	1
1.2	CIMDLs can refer both to sinonasal and palatal lesions due to cocaine snorting	8.83	9	8	9	0	strong consensus	1
1.3	CIMDL diagnosis should not rely on auto-antibody testing, which might result positive or negative results in CIMDL patients	8.50	9	7	9	0	strong consensus	3

**Table 2:** Statements and results from the Delphi process for items reaching consensus or strong consensus: clinical evaluation and diagnosis.

Item No.	Statement	Mean	Median	Min	Max	Outliers	Result	Delphi Round
2.1	Clinical assessment of potential CIMDLs should include face examination, anterior rhinoscopy, oral examination, and nasal endoscopy	8.61	9	7	9	0	strong consensus	1
2.2	The degree of nasal structures involvement can be assessed via the Nitro et al. classification of CIMDLs	8.44	9	5	9	0	consensus	1
2.3	Differential diagnoses for CIMDLs include vasculitis (including but not limited to granulomatosis with polyangiitis), T-cell or NK/T lymphoma, infectious disease (including, but not limited to, syphilis, leishmaniasis, yaws, leprosy, tuberculosis, and actinomycosis), chronic intestinal bowel disease (e.g., Crohn’s disease), chronic use of vasoconstrictor drugs, rhinotillexomania, trauma, other recreational intranasal drug use (amphetamine, acetaminophen–oxycodone), IgG4-related disease, relapsing polychondritis, rhinoscleroma, sclerosing orbital pseudotumor, and iatrogeny	8.50	9	6	9	1	consensus	2
2.4	In the case of isolated septal perforations, cocaine-snorting habits should be investigated in the patient history as well as traumas, prior nasal or nose-involving procedures (including radiotherapy and intranasal oxygen therapy), prolonged intranasal vasoconstrictor use, picking habits or history of foreign bodies, known infectious diseases, and known autoimmune disease or vasculitis	8.67	9	7	9	0	strong consensus	2
2.5	If an isolated septal perforation is not identified as a CIMDL, but no other causative factor can be identified, a biopsy should be considered with the patient, and—if ruled out—a personalized follow-up should be planned to check for lesion evolution, possibly with measurement and clinical image documentation	8.33	9	7	9	0	strong consensus	2
2.6	Patients presenting with a potential CIMDL grade II or higher, without a history of cocaine-snorting, should undergo a complete workup including serology (ESR, ANA, C-ANCA—with immunofluorescence for NPO and PR3 if positive—IgG4, and rheumatoid factor), rheumatology/internal medicine and infectious disease consultation, and nasal biopsy	7.78	9	3	9	1	consensus	1
2.9	In patients presenting with a potential CIMDL without a history of cocaine snorting and with negative workup and cocaine testing results, other less common snorting substances can be assessed (e.g., methamphetamine, heroin)	7.89	8	5	9	1	consensus	1
2.1	Destructive midline lesions due to nasal administration of non-cocaine recreational drugs (e.g., amphetamine, acetaminophen–oxycodone) can be clinically managed as CIMDLs	7.83	9	1	9	1	consensus	3
2.11	Patients presenting with a potential CIMDL and confirming a cocaine-snorting habit should be considered de facto CIMDL patients and receive adequate care and follow-up, though biopsies might still be discussed with the patient to rule out differentials	8.06	9	3	9	1	consensus	3
2.12	Enlarging isolated septal perforations in non-CIMDL patients should undergo a complete workup including serology (ESR, ANA, C-ANCA—with immunofluorescence for NPO and PR3 if positive—IgG4, and rheumatoid factor) and nasal biopsy (for histological and microbiological analysis), considering rheumatology, internal medicine, or infectious disease referral according to results	8.67	9	7	9	0	strong consensus	3
2.13	Patients reporting complete cessation of RNCU with an increase in CIMDL involvement at follow-up should undergo a complete workup including serology (ESR, ANA, C-ANCA, and rheumatoid factor), rheumatology/internal medicine/infectious disease consultation, and nasal biopsy (both for histological and microbiological analysis)	8.56	9	7	9	0	strong consensus	3
2.14	In case of an increase in CIMDL involvement at follow-up in patients reporting stopping recreational cocaine use, a drug test can be considered	8.22	9	5	9	1	consensus	1
2.15	Patients with potential CIMDLs, especially with negative workup, can be offered a nasal mucosal biopsy under local or general anesthesia to assist with diagnosis	7.94	9	3	9	1	consensus	3

**Table 3:** Statements and results from the Delphi process for items reaching consensus or strong consensus: first-line management of CIMDL patients.

Item No.	Statement	Mean	Median	Min	Max	Outliers	Result	Delphi Round
3.1	CIMDL patients could be encouraged to meet a specialist in addiction medicine	8.39	9	3	9	1	consensus	2
3.2	Pain management in CIMDL patients should avoid strong opioids and rely on an addiction medicine service and/or pain medicine services in case of uncontrolled pain potentially requiring strong opioids	8.67	9	7	9	0	strong consensus	3
3.3	CIMDL patients must be advised that the only known method for halting lesion development is ceasing recreational nasal cocaine administration	8.61	9	7	9	0	strong consensus	1
3.4	Patients with CIMDL should be correctly informed that no “safe threshold” of cocaine usage prevents further CIMDL evolution	8.89	9	8	9	0	strong consensus	1
3.5	After proper information, any CIMDL patient can be offered a complete workup including serology (ESR, ANA, C-ANCA—with immunofluorescence for NPO and PR3 if positive—IgG4, and rheumatoid factor), rheumatology, internal medicine and/or infectious disease consultation, and nasal biopsy for increasing compliance to medical advice and therapies	8.22	8.5	7	9	0	strong consensus	3
3.6	Grade II or higher CIMDL patients should always be assessed for signs of rhinosinusitis even in asymptomatic cases	8.50	9	6	9	1	consensus	2
3.7	Grade III or higher CIMDL patients should be counseled for signs and symptoms of orbital or skull base complications and instructed to report to emergency department or specialty services promptly for evaluation	8.17	9	3	9	1	consensus	1
3.8	Potential CIMDL extension and signs of secondary rhinosinusitis should be assessed with a CT scan, with the use of contrast medium limited to cases with suspected osteitis or osteonecrosis	8.00	9	3	9	1	consensus	1
3.9	A baseline plain CT of the sinus (including the orbits and skull base) is recommended in patients presenting with CIMDL stage II or higher	8.56	9	7	9	0	strong consensus	2
3.1	The use of MRI in CIMDLs should be limited to the evaluation of orbital, skull base, intracranial, or other soft tissue involvement	8.44	9	7	9	0	strong consensus	2
3.11	The impact of CIMDLs on sinonasal health should preferably be investigated with general nasal health questionnaires, though sinusitis-specific questionnaires may have a clinical and investigational role	8.39	9	5	9	1	consensus	3
3.12	CIMDL patients should be instructed to employ daily saline nasal lavages for nasal toilette and can employ nasal emollients for reducing crusting	8.44	9	3	9	1	consensus	1
3.13	Antibiotic therapy (systemic and/or topical) should be reserved for bacterial superinfections or osteitis, obtaining bone cultures in the latter scenario whenever possible	8.72	9	7	9	0	strong consensus	3
3.14	Endoscopic debridement of crusting and necrotic tissues during outpatient follow-up and/or high-volume nasal lavages can be offered to improve symptoms such as nasal obstruction and cacosmia	8.72	9	7	9	0	strong consensus	3
3.15	CIMDL patients with palatal perforation can be offered palatal obturator prostheses as first-line management	8.11	9	3	9	1	consensus	1
3.16	Grade IV CIMDL patients should be proposed pneumococcal, haemophilus and meningococcal vaccine administration	8.22	9	3	9	1	consensus	3
3.17	Initial follow-up should be personalized and planned according to the CIMDL stage, considering more frequent visits to encourage RNCU cessation; in stable patients, a 6–12 month follow-up can be proposed up to 2–5 years after stopping cocaine snorting	8.28	9	7	9	0	strong consensus	3

**Table 4:** Statements and results from the Delphi process for items reaching consensus or strong consensus: surgical management of complications and reconstructions.

Item No.	Statement	Mean	Median	Min	Max	Outliers	Result	Delphi Round
4.1	CIMDL patients with symptoms and signs of rhinosinusitis should be proposed adequate medical and/or surgical treatment as per EPOS/ICAR:RS guidelines	8.44	9	5	9	2	consensus	2
4.2	Patients with signs and symptoms of osteitis or osteonecrosis should be offered debridement of necrotic tissue and adequate systemic antibiotic treatment guided by culture biopsy of a bony specimen and planned together with an infectious disease consultation	8.39	9	4	9	1	consensus	2
4.3	In patients with intraorbital complications from CIMDL, culture-guided antibiotic therapy should represent the first-line management, while endoscopic transnasal surgical debridement and drainage should be reserved for cases with sudden visual acuity reduction or loss of color perception, large or superior/lateral orbital abscesses, or failing medical therapy	8.44	9	7	9	0	strong consensus	3
4.4	Grade IV CIMDL without CSF leaks or intracranial complications should be managed conservatively (nasal toilette, antibiotics for superinfections, vaccine recommendarions)	8.72	9	7	9	0	strong consensus	3
4.5	In fit-for-surgery grade IV CIMDL patients with ascertained CSF leaks, thorough debridement of necrotic tissues and prompt skull base reconstruction are recommended, and a neurosurgical consultation should be considered	8.67	9	7	9	0	strong consensus	3
4.6	Patients with intracranial complications should be managed with culture-guided antibiotic therapy and/or surgical toilette via an endonasal or combined endonasal/craniotomy route, in a multidisciplinary rhinological and neurosurgical team, according to the specific complication	8.72	9	7	9	0	strong consensus	2
4.7	Septal, nasal, and palatal reconstructions should not be performed in CIMDL patients unless the patient provides adequate motivation and proves cocaine use stopping by providing toxicological analyses covering at least 12 months	8.61	9	5	9	1	consensus	2

**Table 5:** Statements and results from the Delphi process for items not reaching consensus or strong consensus.

Item No.	Statement	Mean	Median	Min	Max	Outliers	**Result**	**Delphi Round**
1.4	CIMDLs are due to RNCU only, while they are not related to medical nasal cocaine administration	7.61	9	1	9	2	near-consensus	3
2.7	Patients with suspected CIMDLs denying RNCU and with negative workup results should undergo a cocaine drug test for cocaine to adjust follow-up and treatment	7.83	9	3	9	3	no consensus	3
2.8	RNCU can be identified via urinary metabolites (3 to 15 days after use, also according to the administered dose) or hair analysis (up to 3 months after use)	8.28	9	3	9	2	near-consensus	3
4.8	Reconstructions for CIMDL patients should be tailored to the patients; though pedicled flaps and vascularized free flaps usually represent the first option, autografts can still be considered, and allografts are usually adequate for a minority of patients	7.94	9	4	9	2	near-consensus	3

## Data Availability

The data presented in this study are available on request from the corresponding author.

## Abbreviations

The following abbreviations are used in this manuscript:

RCNU	Recreational Cocaine Nasal Use
CIMDL	Cocaine-Induced Midline Destructive Lesions

## Appendix A

Statements of the evolution across the three Delphi consensus rounds. (Blue: strong consensus; green: consensus; yellow: near-consensus; red: no consensus).
